# A blinded observational cohort study of the microbiological ecology associated with pyuria and overactive bladder symptoms

**DOI:** 10.1007/s00192-018-3558-x

**Published:** 2018-02-17

**Authors:** Kiren Gill, Ryoon Kang, Sanchutha Sathiananthamoorthy, Rajvinder Khasriya, James Malone-Lee

**Affiliations:** 1 Research Centre for Nephrology, Division of Medicine, University College London, London, UK; 2 Urogynecology, University College Hospital, London, UK

**Keywords:** OAB, Infection, UTI

## Abstract

**Introduction and hypothesis:**

This study sought to characterise the microbial ecology of the lower urinary tract in patients with symptoms of overactive bladder (OAB) using culture of the urinary urothelial cell sediment. The pathological significance of the microbiome was assessed through its relationship with known urothelial inflammatory markers and patient reported symptoms.

**Methods:**

Adult female patients with OAB symptoms and asymptomatic controls were assessed at 12 study visits scheduled every 4 weeks. At each visit, all participants provided a clean-catch midstream urine (MSU) that was analysed to count white and uroepithelial cells, submitted to standard culture and spun urothelial-cell-sediment culture. Symptoms were assessed using validated questionnaires.

**Results:**

This analysis shows that OAB patients differ consistently from controls, demonstrating differences in bacterial ecology (*t* −4.57, *p* 0.0001), in the microscopic pyuria count (*t* −6.37, *p* 0.0001) and presence of infected urothelial cells (*t* −4.21, *p* 0.0001). The primary outcome measure of bacterial growth [colony-forming units (CFU) ml^−1^] was higher in OAB patients than in controls throughout the 12 months. Data showed a correlation between symptoms and pyuria, with notable urgency correlating with pyuria and epithelial cell shedding. The routine urine cultures (with a threshold of reporting a positive result as 10^5^ CFU/ml) were unable to distinguish OAB patients from controls. However, sediment cultures differed significantly, and there was a correlated increased immune response amongst OAB patients.

**Conclusions:**

This study supports the need to re-examine the OAB phenotype given this association with microbial colonisation.

## Introduction

The relationship between infection, inflammation and the generation of overactive bladder (OAB) symptoms has not been scrutinised in a prospective, controlled study, and our dismissal of an infective association for this symptom group may be compromised by deficiencies in urinalysis on which we rely heavily [1] . Quantitative microbiological bacterial culture remains the reference gold standard in the diagnosis of urinary tract infection (UTI). However, it is now appreciated that in patients with nonacute lower urinary tract symptoms (LUTS), a midstream urine culture (MSU) that does not show ≥10^5^ colony-forming units (CFU) μl^−1^ of a single species of a known urinary pathogen does not exclude significant UTI [2, 3]. There is growing evidence of polymicrobial infections of the urinary tract [4–6]. Traditional culture methods assume, without evidence, dominant pathogenicity from the *Enterobacteriaceae* species, notably *E. coli*, so the MSU culture is performed on selective media, favouring *Enterobacteriaceae* under aerobic conditions. This culture method will miss a number of potential pathogens [3]. Microscopic counting of pyuria remains the best surrogate marker of UTI that we have [7]. However, in some clinical settings, dipstick tests to detect leucocyte esterase have replaced leucocyte counting by fresh-urine microscopic examination, without validation. Despite this, some laboratories use dipsticks to screen urine samples; others use microscopy, only culturing urine samples that are pyuria-positive. Where microscopy is used, the delay caused by specimen transit compromises the specimen [8]. In assessing anyone who presents with OAB, a mandatory step is the exclusion of UTI, but given the deficiencies in the tests, an OAB diagnosis may be flawed.

Studies of patients in the broader category of LUTS report increased inflammatory activity and bacterial colonisation not seen in asymptomatic controls [3, 9]. Bacterial strains from LUTS patients were shown to invade urothelial cell lines, whilst bacteria isolated from controls did not [10]. It is possible that LUTS may be associated with urothelial microbial changes that stimulates an inflammatory response, generating the symptoms [11, 12]. OAB is an important subclass of LUTS that merits specific attention. Lunawat et al. found that in all 61 patients with OAB and pyuria but negative routine microbial culture, bladder biopsies manifested all the uroepithelial features of chronic cystitis; no features of inflammation were identified in control samples [13]. Vijaya et al. found increased bacterial growth on culturing bladder biopsies obtained at cystoscopy from patients with OAB despite negative MSU culture [14].

This blinded study scrutinised patients, specifically those with OAB, comparing them with normal controls and monitoring inflammatory and microbiological activity in the urinary tract over 12 months.

## Materials and methods

### Study groups

Patients were recruited from urological clinics and controls from staff or volunteers. Female patients who described OAB symptoms according to International Continence Society (ICS) criteria [15], of urinary urgency with or without urge urinary incontinence (UUI) were included. Healthy female adults matched for age and menopausal status and with no urinary symptoms formed the control group. All participants provided written informed consent and completed the International Consultation on Incontinence Questionnaire (ICIQ) LUTS for urgency and pain. Women who were pregnant or planning pregnancy were not eligible for inclusion. Participants with other urinary tract disease, diabetes, immune disease or taking diuretics or other drugs influencing the urinary tract, which may have compromised data validity, were excluded.

### Symptom questionnaires

Symptoms were recorded using three validated questionnaires: The ICIQ was selected to evaluate symptoms [16]. The Whittington Urgency Score, a ten-item scale, was used to measure symptoms and degree of urinary urgency; the questionnaire has been validated [17, 18]. The Whittington Pain Questionnaire, a validated, eight-item scale, was used to record the most prevalent dysaesthetic/pain symptoms associated with the lower urinary tract [19].

### Study visits and processes

Written informed consent was obtained at the first visit, prior to any study-related procedures, and eligibility was checked. Participants attended 12 study visits in total, scheduled every 4 weeks. During this time, patients were treated with antimuscarinics agents and antibiotics if pyuria implied infection.

### MSU sample collection

Participants provided a midstream clean-catch urine sample. Patients were given verbal and written instructions on avoiding contamination [20]. The urine was decanted into three 30-ml sterile universal, anonymised specimen tubes, blinding the researcher.

### Inflammation and immune response: microscopy for pyuria and urothelial cell shedding

Immediate microscopy was performed on fresh, unspun, unstained urine samples. A disposable pipette was used to place a drop of urine in the filling chamber of a Neubauer haemocytometer and covered with a glass coverslip. Olympus CX41 light microscope (×200) (Olympus, Southend-on-Sea, UK) was used to analyse the sample. Leucocyte and epithelial cell count was enumerated using a standard operating procedure in triplicate. All three measures were recorded and mean value calculated.

### Bacterial colonisation: urothelial clue-cell analysis

Urine samples were processed within 1 h of collection and refrigerated at 4 °C until assessment. A collection chamber consisted of a single-channel cuvette and retainer, a Shandon filter card (Fisher Scientific, Loughborough, UK) and a Superfrost Ultra Plus glass microscope slide; 80 μl of urine was transferred into the collection chamber for centrifugation and spun at 75 g for 5 min. Cellular components formed a visible deposit on the slide. Cells were then fixed with 4% formaldehyde (Thermo Scientific, Fisher Scientific) at room temperature for 15 min. Cell membranes were stained with wheat germ agglutinin (WGA) conjugated to Alexa Fluor 488 (Invitrogen). The cellular deposit was incubated for 15 min at room temperature. Alexa Fluor 488 excites at a wavelength of 495 nm and emits at 519 nm; hence, the cell membranes appeared green under fluorescent microscopy. The host and bacterial DNA were stained using the DNA stain 4′-6′-diamidino-2-phenylindole (DAPI) and cells immediately mounted with FluorSave reagent (Calbiochem). A coverslip was carefully applied ensuring no air bubbles, and the coverslip was fixed with nitrocellulose with ethyl acetate. DAPI gives mammalian nuclei and bacteria a blue appearance under fluorescent microscopy; it excites at a wavelength of 360 nm and emits at 460 nm. DAPI is able to label intracellular and extracellularly attached bacteria without the need for permeabilisation. Slides were examined under a fluorescent Olympus CX41 upright epi-fluorescence microscope. The proportion of clue cells—urothelial cells exhibiting adherent or intracellular microbes—were calculated by counting the total number of cells present and then the proportion of cells with associated bacteria. Counts were performed in triplicate, and an average clue-cell proportion was recorded.

### Microbiological assessment: enhanced sediment culture

Enhanced sediment cultures were processed within 2 h of sample collection. Samples were refrigerated at 4 °C until processed. Five millilitres of fresh unspun, unstained, urine was centrifuged at 627 g for 5 min in a Denley BR401 centrifuge (R_MAX_ 140 mm) (Denley, Heckmondwike, UK). The supernatant was removed, leaving the urinary sediment, which was resuspended in 400 μl of 1% sterile phosphate-buffered saline (PBS) solution. Four 1:10 serial dilutions were performed for accurate quantitative bacterial counting. Chromogenic CPS3 agar plates (bioMérieux, Basingstoke, UK) were used for culture. All culture plates were incubated aerobically for 24 h at 37 °C in a CO_2_-dependant incubator.

#### Bacterial quantification

Each bacterial isolate was quantified. No threshold was used to discriminate positive or negative growth. The mean colony count from all sectors was calculated. The CPS3 chromogenic medium allows bacterial identification of uropathogens to genus or species level, dependent on the microbe. The growth of distinct bacteria is colour-specific to allow easy and fast enumeration. Colour identification was based on the manufacturer’s standardised colour guide and supplemented with Gram staining and rapid biochemical tests for further characterisation. Analytical Profile Index (API) testing was used when these methods were unable to identify an isolate conclusively.

### Microbiological assessment: routine culture

All routine microbiological cultures were undertaken in the hospital microbiology laboratory. Thirty millilitres of urine in a sterile universal specimen tube was cultured immediately upon receipt or after overnight refrigeration at 4 °C. Trained blinded biomedical scientists undertook all analyses. One microlitre of the urine sample was inoculated onto a CPS3 agar plate using a sterile 1-μl loop. The culture plate was then incubated aerobically for 24 h at 37 °C. Bacterial colonies were identified by colour and morphologic characteristics. Rapid reagent testing (spot testing) was employed to supplement colour-based bacterial identification. Bacterial growth was estimated by visual assessment of colony density. A positive culture was defined as the growth of a single recognised uropathogen at ≥10^5^ CFU ml^–1^. Polymicrobial growth above this threshold was reported as mixed growth. Any bacterial growth <10^5^ CFU ml^–1^ was reported as no significant growth.

### Primary and secondary outcome measure

The primary outcome measure was total log_10_ bacterial CFU of all isolates obtained from culture of the urinary sediment. The secondary outcome measures were:

- Microscopic pyuria count
- Urothelial cell count
- Urothelial cells demonstrating associated bacteria (clue cells)
- Routine urine culture in hospital laboratory
- ICIQ-LUTS symptoms score
- Whittington Urgency Score
- Whittington Pain Score

### Statistical analysis

A sample size of 20 participants in each group provided 83% power to detect a significant difference in log_10_ bacterial growth, with alpha of 0.05. This was calculated from pilot data in which the log_10_ bacterial count standard deviation (SD) was 2 and mean difference 0.5. The primary analysis was to determine the difference in total log_10_ CFU per ml^−1^ of bacterial growth between patients and controls. The independent variable was group (patient 1, control 0), which was entered as a fixed effect. The dependant variable was total bacterial growth on sediment culture (log_10_ CFU ml^−1^). Measure repetition was identified by visit number.

Secondary analyses were explored the relationship between bacterial growth, LUTS, pyuria and urinary urothelial cell shedding. With the independent variable being group (patients/controls), dependent variables were selected, in turn, as LUTS, urgency and pain scores, log_10_ pyuria and log_10_ epithelial cell count (μl^−1^). Measure repetition was identified by visit number.

For additional analysis, data were pooled into two sets, patients and controls, and pooled to compare the performance of routine culture methods against results of urinary sediment culture. Analyses of monthly data were achieved through the repeated-measures procedure of the generalised linear model (GLM) provided by SPSS. The nonparametric Mann–Whitney test was used to examine pooled data for differences in outcomes between patients and controls. Multinomial logistic regression was used to examine differences in microbial species dispersion between groups.

## Results

Between April 2011 and September 2013, 24 female patients with OAB (mean age 63 years; SD 11) and 22 asymptomatic controls (mean age 59 years; SD 12) were recruited. Patients had a mean symptom duration of 1.3 years. Both groups were matched for menopausal status and body mass index (BMI) (Table 1). There was one dropout from each group. Results are from the pooled data analysis of 282 patient and 253 control visits.

**Table 1:** Demographics

Parameter	Patients, mean (SD)	Controls, mean (SD)	*P* value
Age (years)	63 (11)	59 (12)	0.13
Body mass index	28 (5.3)	26 (5.4)	0.11
Menopausal status	Pre 3, post 21	Pre 3, post 19	0.65

*SD* standard deviation, *WBC* white blood cells

These analyses showed significant differences between patients and controls in bacterial load (log_10_ CFU ml^−1^). There were also differences in total symptoms, pain and urgency, pyuria (log_10_), WBC (μl^−1^), epithelial cell shedding (log_10_ endothelial progenitor cells (EPC) μl^−1^) (Table 2).

**Table 2:** Linear mixed-effects model analysis using group as the fixed effect

Dependent variable	Parameter estimate^a^	Significance
Bacterial growth^b^	−1.08 (95% CI −1.55 to −0.60; *t* −4.57; *df* 41.3)	*P* 0.000
LUTS score	−16.11 (95% CI −19.1 to −13.1; *t* −10.8; *df* 43.8)	*P * 0.0001
Urgency	−6.95 (95% CI −8.65 to −5.24; *t* −8.21; *df* 44.1)	*P*0.0001
Pain	−1.44 (95% CI −2.23 to −0.64; *t* −3.65; *df* 44.1)	*P * 0.0001
Pyuria count^c^	−0.57 (95% CI −0.75 to −0.37; *t* −6.37; *df* 41.7)	*P* 0.0001
Epithelial count^d^	−0.30 (95% CI −0.44 to −0.15; t −4.21; *df* 43.1)	*P* .00001

*CI* confidence interval, *t* distribution, *df* degrees of freedom, *CFU* colony-forming units, *WBC* white blood cells, *EPC* endothelial progenitor cells

^a^Parameter estimate: increase in magnitude of dependent variable demonstrated by controls compared with patients

^b^Bacterial growth: log_10_ CFU ml^−1^

^c^Pyuria count: WBC ul^−1^

^d^Epithelial count: EPC ul^−1^

Since pyuria is the best surrogate marker of infection that we have [7, 8], the relationship between outcome measures and pyuria were examined using the linear mixed-effects model procedure to achieve a multiple regression, with independent variables selected as total microbial growth (log_10_ CFU ml^−1^); group; total LUTS, urgency and pain scores; and epithelial cell shedding (log_10_ EPC μl^−1^) (Table 3). The dependant variable was pyuria (log_10_ WBC ml^−1^). Visit number identified the repeated measures. Variables that were discriminating for log_10_ pyuria were log_10_ bacterial growth, group number, total LUTS score and log_10_ epithelial cell shedding.

**Table 3:** Multiple mixed models analysis with log_10_ pyuria as the dependant variable

Parameter	Parameter estimate^a^	Significance
Bacterial growth^b^	0.14 (95% CI = 0.17 to 0.11; *t* = 8.96; *df* = 483.8)	*p* = .0001
Group	−0.35 (95% CI = −0.09 to −0.54; t = −4.76; df = 47.5)	*p* = .0001
LUTS score	0.13 (95% CI = 0.02 to 0.01; t = 2.89; df = 363.6)	*p* = .0001
Epithelial count^c^	0.25 (95% CI = −0.35 to −0.15; t = 5.10; df = 474.9)	*p* = .0001

*CI* confidence interval, *t* distribution, *df* degrees of freedom

^a^Parameter estimate: Increase in magnitude of dependent variable demonstrated log_10_ pyuria

^b^Bacterial growth: log_10_ cfu ml^−1^

^c^Epithelial count: epc ul^−1^

**Fig. 1:**
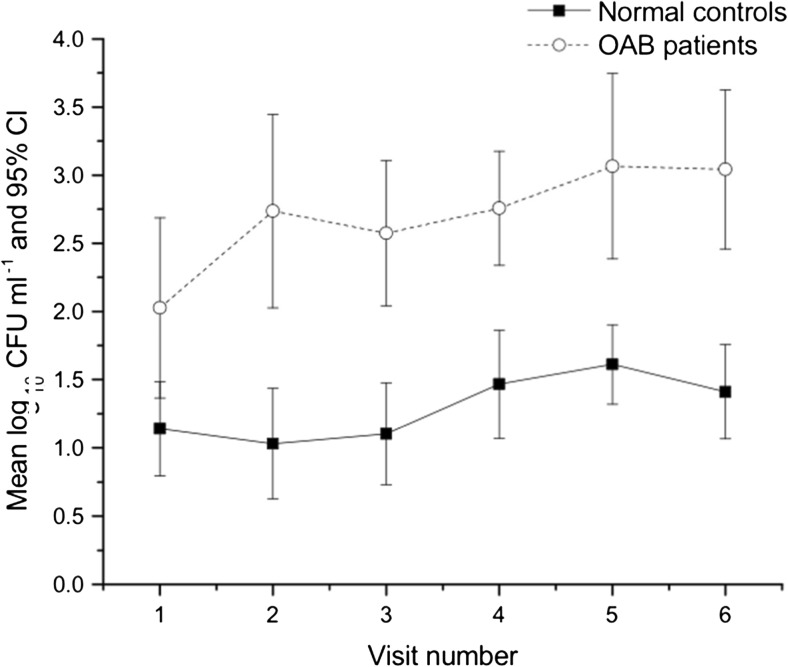
Mean log_10_ bacterial growth in patients and controls at each visit

**Fig. 2:**
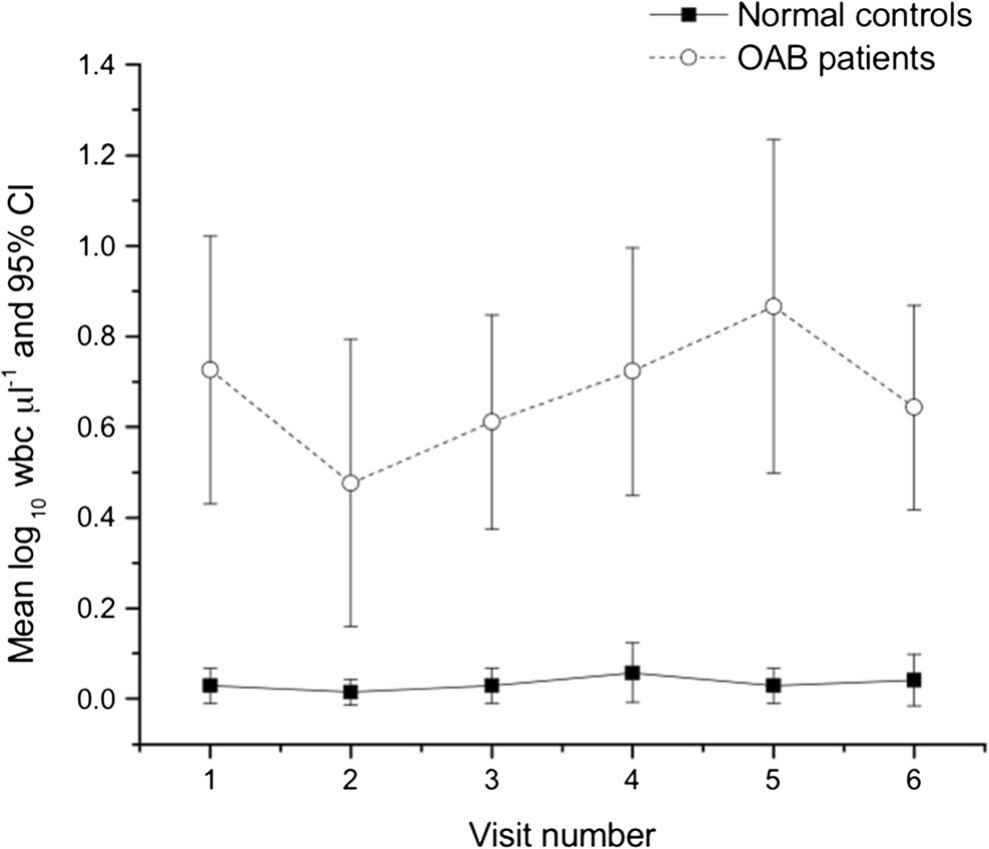
Mean log_10_ pyuria in patients and controls at each visit

At each visit, patients consistently showed a higher log_10_ bacterial growth compared with controls on spun sediment culture (Fig. 1). The same applied to pyuria on fresh urine microscopy (Fig. 2).

**Fig. 3:**
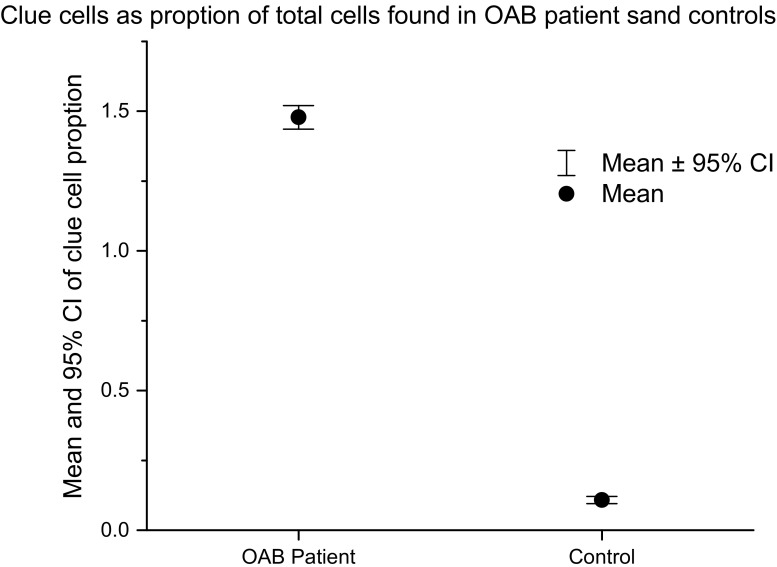
Proportion of clue cells found in patients and controls

Pooled analysis of the spun sediment culture showed significantly greater bacterial growth in patients than in controls (*Z* −5.981, *p* 0.0001). Median log_10_ total colony counts in the patient group were 2.30 CFU ml^−1^ [interquartile range (IQR)1.86–3.84) compared with 1.50 CFU ml^−1^ (IQR 0.13–1.61) in the control group. The patient group also showed significantly greater clue-cell shedding (Fig. 3) (β 1.48, *df* 1, *p* .0001). In the control group mean clue-cell proportion was 0.01, median 0.00 (SD 0.057, IQR 0.0–0.08) and in the patient group 0.19, median 0.17 (SD 0.16, IQR 0.11–0.26).

**Fig. 4:**
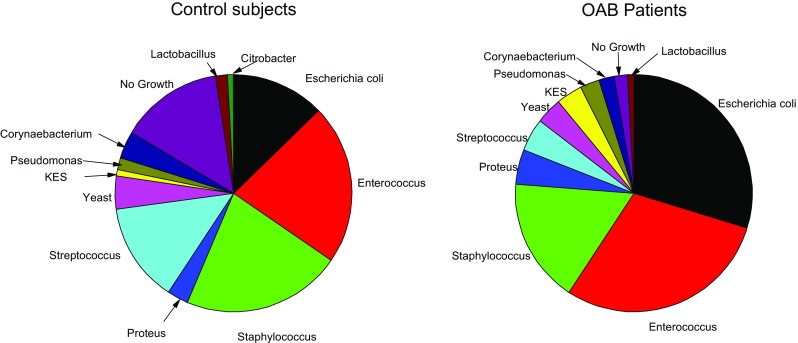
Microbial diversity in patients and controls

**Fig. 5:**
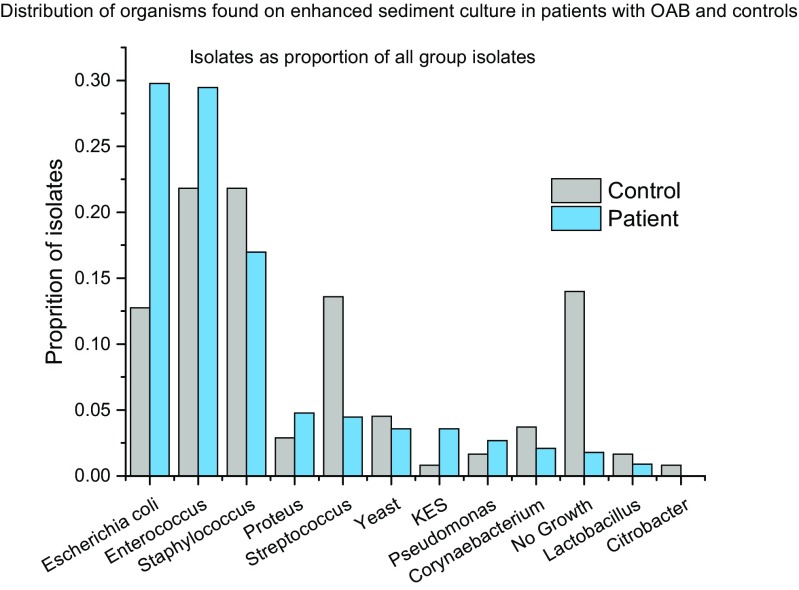
Distribution of organisms found on enhanced sediment culture in patients and controls

**Fig. 6:**
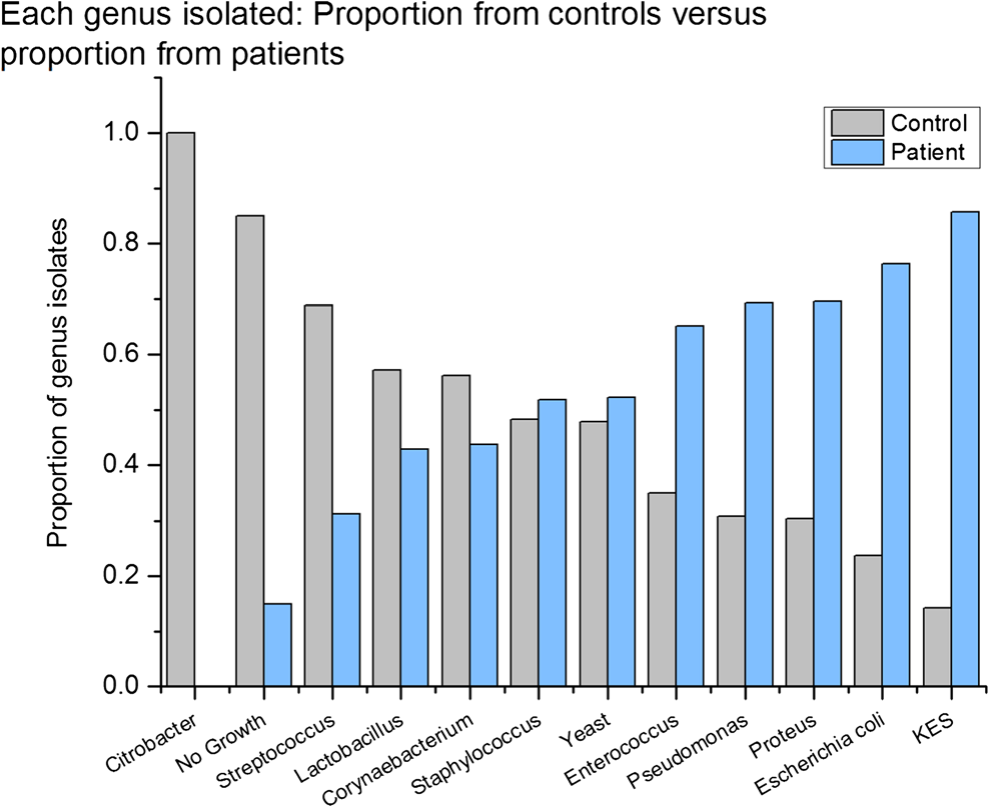
Proportionate distribution between patients and controls of each organism genus

Two patients had a positive routine culture at some stage during the year compared with one in the control group; 93.4% (*n* 265) of patients grew microbes from spun sediment culture (> 0 CFU ml^−1^), which was significantly higher than in controls, where 70.1% (*n* 197) grew microbes (χ^2^ 51.33, *p* 0.0001). Microbial diversity was distinctly different between patients and controls. In patient cultures, recognised uropathogens predominated. Using multinomial logistic regression with microbes entered into the model as factors and dependant variable being group, *E. coli* and other coliforms and no growth proved significant group discriminators (χ*2* 82.8, *p* 0.0001). Figures 4 and 5 show the distribution of organisms isolated from patients and controls. Figure 6 shows a proportionate occurrence between controls and patients of each organism genus; thus, column pairs add to 1 in each case. There is a distinct variation in bacteria between groups; bacterial genus found in some patients occurred less frequently in controls.

## Discussion

This study has demonstrated differences in bacterial ecology between OAB patients and controls over 12 monthly assessments. Groups contrasted in surrogate and direct markers of inflammation and infection. Whilst there was an improvement in patient symptoms, scores never fell to those of the control levels. At this time, the differences between groups are novel observations that demand further observation and explanation; the pathophysiology of OAB needs fresh scrutiny.

The ICS definition of OAB is clear and easy to apply, and 24 patients met that definition. There is significant literature that challenges the accuracy of routine urine culture and screening tests calibrated to this. The OAB diagnosis is predicated on the exclusion of UTI, but the tools used to do this are lacking. Data from this study imply that we are too complacent in dismissing infection in the aetiology of OAB, and this phenotype merits revalidation.

In this study, routine laboratory culture did not differ between patients and controls at any stage. It is worrying that the gold standard diagnostic test cannot discriminate patients from controls, despite other measures showing clear, consistent, inflammatory and microbiological differences. Regression analysis found pain to have some discriminating properties, and the bladder microbiome has been explored in patients with painful bladder syndrome [21].

Despite the dropouts, we maintained statistical power throughout. Groups were matched for key demographics of age, menopausal status and BMI. The common bacterial isolates found on spun sediment in patients were different from the predominant bacterial isolates from controls. This is commensurate with data reported by Khasriya et al. [22]. *E. coli* was the most prevalent species amongst patients, followed by *Enterococcus faecalis*. *Proteus*, KES group (*Klebsiella sp., Enterobacter sp. and Serratia sp.*) and *Pseudomonas* were also found more commonly in the patient group. Amongst controls, *Staphylococcus*, *Streptococcus*, *Citrobacter* and *Lactobacillus* were more commonly found. The observation of *E. coli* as the most prevalent organism amongst patients echoes similar studies of acute UTI [23]. The second-most prevalent bacterium in patients was *E. faecalis*. This, too, is a recognised urinary pathogen, and recently, Horsley et al. [24] showed good evidence of intracellular colonisation of urothelial cells by *E. faecalis* using confocal microscopy.

Most spun sediment cultures from patients and some from controls demonstrated polymicrobial growth. Whilst mixed-growth cultures historically have been blamed on contamination from poor sampling, this has been strongly challenged [25, 26]. Wolcott et al. suggested that coexistence of certain species of bacteria, resulting in polymicrobial cultures, may offer microbes a survival advantage [27]. Our study did not address this hypothesis.

Urothelial clue-cell shedding has been described as an immune response to infection, with murine and human studies showing increased cell shedding in response to infection [28, 29]. This study found that the patient group showed increased proportions of urinary clue cells, which are urothelial cells manifesting adherent or intracellular microbes. The increased clue-cell proportions imply increased microbial colonisation of urothelial cells in OAB patients. This observation is in line with other reports [30].

In considering sources of error, the measurement of bacterial in spun urothelial cells deposits could have been affected by the sampling method. Bacteria from the lower genital tract could have contributed to this. However, the immediate refrigeration of urine and the processing of these samples within 1 h of collection reduced this risk. Whilst one could argue that the MSU sampling method could contribute to contamination, this would appear to be nominal given the low pyuria counts and urothelial cell counts in the control population.

We did not use a quantitative threshold to define significance in a spun sediment culture or apply the criterion of a single species. There are no data to guide such thresholds, so we report data without categorisation. There are quantitative differences in microbial isolates, but we are in no position to state what those mean. Analysis of urothelial and clue cells is novel, as is sediment culture, but these methods have been well validated and have a strong pathophysiological foundation. Fresh urine microscopy is not commonly adopted in clinical practice nowadays, nevertheless, it has been well validated in studies dating back to 1928 and is still unsurpassed as a surrogate marker of infection [7, 8]. DNA sequencing may be an alternative, although it still lacks quantitative specificity.

We did not take account of urine concentration, and there was no diurnal control of sampling. Patients were not asked to alter fluid intake at the time of sampling. Patients with pyuria were treated for infection, but response was slow. This may be indicative of another well-described problem: We now know that a UTI, if untreated, can lead to parasitisation of urothelial cells with microbes that commonly protect themselves in biofilms. Such long-term infections can be exceedingly hard to eradicate [31, 32]. Patients had symptoms untreated with antibiotics for a mean of 1.3 years. Additionally, the normal bladder is far from sterile and hosts a significant microbiome, so we should not necessarily expect treatment to result in microbial decolonisation [32, 33].

This study shows consistent, reproducible and sustained differences between OAB patients and controls in the microbial load contained in a urinary spun sediment, dispersion of isolated species, pyuria, urinary urothelial cell shedding and clue-cell excretion. The latter three are well-validated surrogate indicators of infection [7, 8, 24]. It would seem that alterations in the bladder microbiome might play a significant role in OAB, particularly as symptoms correlated with the inflammatory markers.
